# Assessment by the Scheimpflug imaging system of corneal clarity and anterior segment properties in rosacea patients^⋆^

**DOI:** 10.1016/j.abd.2023.07.005

**Published:** 2024-04-12

**Authors:** Erman Bozali, Duygu Yalınbaş Yeter, Mustafa Tosun, Anıl Selim Apa

**Affiliations:** a Department of Ophthalmology, Cumhuriyet University Faculty of Medicine, Sivas, Turkey; b Department of Dermatology, Cumhuriyet University Faculty of Medicine, Sivas, Turkey; c Department of Ophthalmology, Ministry of Health Isparta City Hospital, Isparta, Turkey

**Keywords:** Cornea, Corneal wavefront aberrations, Densitometry, Dry eye syndrome, Rosacea

## Abstract

**Objective:**

To evaluate the effects of rosacea on ocular surface changes such as alterations in dry eye parameters, corneal densitometry, and aberrations, in comparison with healthy controls.

**Methods:**

A total of 88 eyes of 44 patients diagnosed with rosacea and 88 eyes of 44 healthy controls were enrolled in this cross-sectional study. All participants underwent a comprehensive dermatologic and ophthalmic examination and Tear Break-Up Time (TBUT) and Schirmer-1 tests were performed. The rosacea subtype and Demodex count and OSDI scores of all participants were recorded. Corneal topographic, densitometric, and aberrometric measurements were obtained using the Scheimpflug imaging system.

**Results:**

The mean age of the 44 patients was 41.2 ± 11.0 years of whom 31 (70.5%) were female. The mean TBUT and Schirmer-1 test values were significantly decreased and OSDI scores were significantly increased in the rosacea group compared to healthy controls (p < 0.01 for all). The most common subtype of rosacea was erythematotelangiectatic rosacea (70.4%). The severity grading of rosacea revealed that 18 (40.9%) patients had moderate erythema. The median (min-max) Demodex count was 14.0 (0–120) and the disease duration was 24.0 (5–360) months. The comparison of the corneal densitometry values revealed that the densitometry measurements in all concentric zones, especially in central and posterior zones were higher in rosacea patients. Corneal aberrometric values in the posterior surface were also lower in the rosacea group compared to healthy controls. The topographic anterior chamber values were significantly lower in the rosacea group.

**Study limitations:**

Relatively small sample size, variable time interval to hospital admission, and lack of follow-up data are among the limitations of the study. Future studies with larger sample sizes may also enlighten the mechanisms of controversial anterior segment findings by evaluating rosacea patients who have uveitis and those who do not.

**Conclusion:**

Given the fact that ocular signs may precede cutaneous disease, rosacea is frequently underrecognized by ophthalmologists. Therefore, a comprehensive examination of the ocular surface and assessment of the anterior segment is essential. The main priority of the ophthalmologist is to treat meibomian gland dysfunction and Demodex infection to prevent undesired ocular outcomes.

## Introduction

Rosacea is a chronic progressive disease that causes inflammation on the skin and the ocular surface with a relapsing and remitting course.^1^ Classification of rosacea consists of four subtypes (erythematotelangiectatic, papulopustular, phymatous, and ocular) and one variant (granulomatous) regarding primary and secondary features described by the National Rosacea Society Expert Committee on the Classification and Staging of Rosacea.^2^ The disease is usually seen in middle-aged populations with a slight female predominance and of unknown origin, however, an innate immune system, dysbiosis, or variations in microbiome including Demodex infestation may play a role in the inflammatory cutaneous response.^3^, ^4^, ^5^ Although the disease is considered one of the most well-known chronic inflammatory conditions of the skin, ocular involvement may be present in 58%–72% of patients causing eyelid and ocular surface inflammation.^6^ However, severe ocular rosacea is generally underappreciated despite causing vision loss when left untreated or inadequately treated.^7^

The ocular manifestations of rosacea due to chronic external ocular inflammation are often overlooked by ophthalmologists and can occur with severe, mild, or even absent cutaneous manifestations.^7^ Ocular involvement is bilateral and commonly affects eyelids, conjunctiva, and cornea. Blepharitis, meibomian gland dysfunction, dry eye disease (DED), recurrent hordeola/chalazia, and telangiectasias of the lid margin are the predominant manifestations of the eyelids.^8^ Diffuse hyperemia with marked congestion of the bulbar conjunctival vessels in the interpalpebral space is common and chronic conjunctivitis may lead to cicatrization of the conjunctiva. The presence of superficial punctate keratitis that is typically observed on the lower third of the cornea, neovascularization, infiltrates, phlyctenules, edema, peripheral pannus, corneal thinning, and ulceration are among the corneal findings of the disease.^9^ Also, previous studies reported biomechanical alterations due to increased proteolytic activity and inflammation on the ocular surface.^10^ Episcleritis, scleritis, and anterior uveitis have also been reported before.^11^ Diagnostic, major, and secondary features of rosacea are presented in Table 1.

**Table 1:** Diagnostic criteria for rosacea.

Diagnostic^a^	Major^b^	Secondary
Fixed centrofacial erythema in a characteristic pattern that may periodically intensify	Flushing	Burning sensation
	Papules and pustules	Stinging sensation
	Telangiectasia	Edema
	Ocular manifestations	Dryness
Phymatous changes	Lid margin telangiectasia	Ocular manifestations
	Interpalpebral conjunctival injection	“Honey crust” and collarette accumulation at the base of the lashes
	Spade-shaped infiltrates in the cornea	Irregularity of the lid margin
	Scleritis and sclerokeratitis	Evaporative tear dysfunction (rapid tear breakup time)

a These features by themselves are diagnostic of rosacea.

b Two or more major features may be considered diagnostic.

Scheimpflug imaging and densitometry are techniques that enable further evaluation of the anterior segment including corneal clarity and light scattering.^12^ Various factors including optical aberrations, diffraction, and light scatter limit the optical quality of the human eye.^13^ The backward light scatter indicates the optical health of the cornea regarding corneal transparency and clarity and thus may be considered a substantial and convenient indicator in the analysis of numerous corneal diseases with repeatability and reproducibility.^14^ Alterations in corneal densitometry and aberrometry in dry eye patients were endorsed in previous studies.^15^, ^16^, ^17^

The aim of this study is to evaluate the effects of rosacea on ocular surface changes such as alterations in dry eye parameters, corneal densitometry, and aberrations, and compare them with healthy controls.

## Methods

A total of 88 eyes of 44 patients diagnosed with rosacea and 88 eyes of 44 healthy controls were enrolled in this cross-sectional study. The control group which consisted of healthy participants without any systemic diseases was paired to the rosacea group by age and gender. The study was approved by the local ethics committee and adhered to the tenets outlined in the Declaration of Helsinki. Written informed consent was obtained from all patients. Participants who had a history of systemic and autoimmune diseases, ocular surgical interventions, and ocular diseases such as glaucoma, corneal ectatic disorders, uveitis, or retinopathy were excluded from the study. A history of trauma, contact lens or topical ocular medication usage, presence of corneal scars, opacities, and conjunctival degenerations such as pterygium were also acknowledged as exclusion criteria. The diagnosis and classification of rosacea were made by the same dermatology specialist (MT) who also evaluated the presence of demodicosis. Classification of rosacea was performed according to the guidelines described by the National Rosacea Society Expert Committee on the Classification and Staging of Rosacea. The patient’s global assessment was graded as absent, mild, moderate, or severe (0–4) which is demonstrated in Table 2.

**Table 2:** Demographics and dry eye parameters of the study population.

	Rosacea (n = 44)	Healthy control (n = 44)	p-value
Age, years	41.2 ± 11.0	38.4 ± 9.6	0.24^a^
Gender (Female, %)	31 (70.5%)	31 (70.5%)	1.0^b^
TBUT test, sec	7.8 ± 3.7	10.9 ± 3.3	<0.001^a^
Schirmer-1 test, mm	14.1 ± 7.0	19.3 ± 7.4	<0.001^a^
OSDI score	23.7 (0–93)	10.2 (0–35)	<0.001^c^
Clinical subtype, n (%)			
*Papulopustular*	13 (29.6%)	‒	‒
*Erythematotelengiectatic*	31 (70.4%)	‒	‒
Systemic treatment, n (%)			
*Tetracycline*	9 (20.5%)	‒	‒
Topical treatment, n (%)			
*Metronidazole*	22 (50%)	‒	‒
*Sodium sulfacetamide*	1 (2.3%)	‒	‒
*Azelaic acid*	1 (2.3%)	‒	‒
Severity, n (%)			
*Absent*	1 (2.3%)	‒	‒
*Mild erythema*	23 (52.2%)	‒	‒
*Moderate erythema*	18 (40.9%)	‒	‒
*Severe erythema*	2 (4.6%)	‒	‒
Demodex count [median(min-max)]	14.0 (0–120)	‒	‒
Disease duration, months [median (min-max)]	24.0 (5–360)	‒	‒

TBUT, Tear Break Up Time; OSDI, Ocular Surface Disease Index.

a Independent sample *t*-test.

b Chi-Square test.

c Mann-Whitney *U*.

A comprehensive ophthalmologic examination including Best-Corrected Visual Acuity (BCVA) using the Snellen chart, intraocular pressure measurement using an air-puff tonometer, slit-lamp biomicroscopy to observe any corneal pathology, and a detailed fundoscopic examination was performed. OSDI scores of all participants were recorded. The ocular surface was assessed with Tear Break-Up Time (TBUT) test and Schirmer-1 tests which are performed by the same experienced clinician who was blinded to the diagnosis and study objective. The Schirmer-1 test was performed without anesthesia utilizing a precalibrated filter paper strip (Schirmer Tear Test Strips, ERC Sağlık, Ankara, Turkey) which is placed on one-third of the lateral portion of the lower eyelid requesting the patients to gently close their eyes without squeezing for 5-minutes. The paper was then removed and the amount of wetting was recorded in millimeters. Tear break-up time was measured using a fluorescein strip paper (Fluorescein Sodium Strips, ERC Sağlık, Ankara, Turkey) wetted with saline and then applied to the inferior bulbar conjunctiva. Patients were asked to blink 3–5 times to form a film over the corneal surface and then not to blink while the tear film was observed under a broad beam of cobalt blue illumination. The TBUT was recorded as the seconds that elapsed between the last complete blink and the appearance of the first dry spot in the tear film. The average of three consecutive measurements was recorded for both tests.^13^

Corneal topographic, densitometric, and aberrometric measurements were performed using a Scheimpflug imaging system (Pentacam HR, Oculus GmbH, Wetzlar, Germany). This imaging system provides a three-dimensional model of the anterior segment consisting of corneal elevation maps, power calculations, and pachymetric and biometric evaluation of the anterior segment using a 180-degree rotating Scheimpflug camera and static camera with a monochromatic slit light source. In line with the device manual, best-aligned and fixated scans with the quality specification examination of “OK” were included for the analysis following three consecutive scans for each eye. Keratometric values (K1, K2, Kmax), thinnest Corneal Thickness (CT), Corneal Volume (CoV), Anterior Chamber Volume (ACV), Anterior Chamber Depth (ACD), Anterior Chamber Angle (ACA), corneal aberrometric measurements including the Root Mean Square (RMS) of total aberrations (RMS-total), RMS of Higher-Order Aberrations (RMS-HOA), RMS of Lower-Order Aberrations (RMS-LOA), and spherical aberrations were calculated from the central 6-mm optical zone with Pentacam’s built-in software v1.25r15. Corneal densitometry was measured automatically with the built-in analysis software provided with Pentacam HR in four concentric zones over a 12-mm corneal diameter. The first zone consists of a circular area with a diameter of two mm in the center of the cornea, and the second, third, and fourth zones are annular areas surrounding the center of 2–6 mm, 6–10 mm, and 10–12 mm, respectively. This analysis also provides densitometric values of the cornea at three different depths: the anterior (superficial 120 μm), central (subtraction of the anterior and posterior layer thickness from total), and posterior (60 μm of the innermost cornea) corneal layers. Corneal densitometry values are expressed as the pixel luminance per unit volume in the Scheimpflug image and are expressed in Grayscale Units (GSU). The light backscatter of the cornea varies from zero GSU meaning no corneal haze to 100 GSU defined as a completely opaque cornea.^18^ All corneal measurements were performed on the same day under the same environmental properties in the same dim-lit room with constant light, temperature, humidity, and airflow setting to prevent possible ocular stress. The Schirmer-1 and TBUT tests were performed after Pentacam HR measurements.

### Statistical analysis

Analyses were performed using Statistical Package for the Social Science (SPSS version 20.0. Armonk, NY: IBM Corp.) software for Windows. The Shapiro-Wilk test was used for the normality of variables. Mean ± standard deviation was provided for normally distributed variables and median (min‒max) was provided for variables that were not normally distributed. The Chi-Square test was used to compare the categorical variables. The Student’s *t*-test or the Mann-Whitney *U* test was used to compare continuous variables according to the data distribution. A value of p < 0.05 was considered statistically significant.

## Results

The mean age of 44 patients was 41.2 ± 11.0 years of whom 31 (70.5%) were female. The distributions regarding age and gender were similar between groups (p > 0.05 for both). The mean TBUT and Schirmer-1 test values were significantly decreased and OSDI scores were significantly increased in the rosacea group compared to healthy controls (p < 0.01 for all). The most common subtype of rosacea was erythematotelangiectatic rosacea (70.4%). The severity grading of rosacea revealed that 18 (40.9%) patients had moderate erythema. Half of the rosacea group was using topical metronidazole ointment and 9 (20.5%) patients were using oral tetracycline. Only 4 patients (9.1%) were using sunscreen. The median (min-max) Demodex count was 14.0 (0–120) and the disease duration was 24.0 (5–360) months. Demographics, patient characteristics, and treatment modalities are summarized in Table 2. The best-corrected VA was 20/20 in both groups. The mean CT was 541.0 ± 33.0 in affected eyes and 533.6 ± 32.8 in healthy fellow eyes and was not statistically significant (p = 0.139). The comparison of the corneal densitometry values revealed that the densitometry measurements in all concentric zones, especially in central and posterior zones were higher in rosacea patients (Table 3). Corneal aberrometric values in the posterior surface were also lower in the rosacea group compared to healthy controls (Table 4). The ACV, ACD, and ACA values were significantly lower in the study population.

**Table 3:** Corneal densitometry measurements of the study population.

	Rosacea (n = 44)	Healthy control (n = 44)	p-value^a^
**Anterior (120 μm) (GSU)**	24.43 ± 5.93	23.49 ± 1.67	0.15
0‒2 mm	22.36 ± 5.66	21.18 ± 1.52	0.06
2‒6 mm	25.94 ± 7.98	23.14 ± 4.88	0.006
6‒10 mm	35.33 ± 10.77	35.19 ± 9.10	0.9
10‒12 mm	26.04 ± 5.76	24.57 ± 2.99	0.03
Total (0–12) mm	24.43 ± 5.93	23.49 ± 1.67	0.15
**Center (GSU)**			
0‒2 mm	15.49 ± 1.72	14.89 ± 0.90	0.004
2‒6 mm	14.00 ± 1.17	13.42 ± 0.69	<0.001
6‒10 mm	17.59 ± 4.86	15.62 ± 3.53	0.002
10‒12 mm	22.67 ± 5.75	22.29 ± 4.50	0.6
Total (0–12) mm	16.80 ± 2.54	15.83 ± 1.70	0.003
**Posterior (60 μm) (GSU)**			
0‒2 mm	11.29 ± 1.24	10.90 ± 0.81	0.015
2‒6 mm	10.81 ± 1.20	10.19 ± 0.75	<0.001
6‒10 mm	14.90 ± 3.67	13.18 ± 2.76	0.001
10‒12 mm	18.70 ± 4.64	18.14 ± 3.82	0.3
Total (0–12) mm	13.51 ± 2.17	12.59 ± 1.56	0.002
**Total thickness (GSU)**			
0‒2 mm	17.02 ± 2.21	16.42 ± 0.84	0.018
2‒6 mm	15.71 ± 2.14	14.94 ± 0.79	0.002
6‒10 mm	19.58 ± 5.13	17.40 ± 3.69	0.002
10‒12 mm	25.57 ± 6.67	25.21 ± 5.15	0.6
Total (0–12) mm	18.77 ± 3.10	17.64 ± 1.93	0.004

GSU, Grayscale Units.

a Independent sample *t*-test.

**Table 4:** Comparison of corneal topographical, aberrometric values and anterior segment parameters of the study population.

	Rosacea patients (n = 44)	Healthy control (n = 44)	p-value^a^
K1 (diopters)	42.92 ± 1.52	43.02 ± 1.42	0.6
K2 (diopters)	43.49 ± 3.56	43.93 ± 1.49	0.2
Kmax (diopters)	44.45 ± 1.75	45.03 ± 2.84	0.1
CT (µm)	541.03 ± 33.07	533.65 ± 32.81	0.1
CoV (mm^3^)	60.20 ± 3.28	60.18 ± 3.43	0.9
ACV (mm^3^)	147.09 ± 42.54	164.34 ± 38.64	0.005
ACD (mm)	2.74 ± 0.39	3.23 ± 0.52	<0.001
ACA (degrees)	32.31 ± 6.95	36.11 ± 5.48	<0.001
RMS Total (front)	1.71 ± 0.80	1.67 ± 0.83	0.7
RMS LOAs (front)	1.59 ± 0.75	1.61 ± 0.81	0.8
RMS HOAs (front)	0.48 ± 0.36	0.42 ± 0.20	0.2
SA (front)	0.26 ± 0.09	0.28 ± 0.06	0.1
RMS Total (back)	0.76 ± 0.16	0.86 ± 0.26	0.003
RMS LOAs (back)	0.74 ± 0.16	0.84 ± 0.25	0.002
RMS HOAs (back)	0.17 ± 0.03	0.19 ± 0.07	0.06
SA (back)	−0.13 ± 0.02	−0.15 ± 0.03	<0.001

K1, Flat keratometry value; K2, Steep keratometry value; Kmax, Maximum Keratometry value; CT, Thinnest Corneal Thickness; CoV, Corneal Volume; ACV, Anterior Chamber Volume; ACD, Anterior Chamber Depth; ACA, Anterior Chamber Angle; RMS, Root Mean Square; LOAs, Low Order Aberrations; HOAs, High Order Aberrations; SA, Spherical Aberrations.

a Independent sample *t*-test.

## Discussion

In this current prospective study, the authors evaluated the effects of ocular surface changes such as alterations on dry eye parameters, anterior segment properties, corneal densitometry, and aberrations that may arise from rosacea. The results of the study demonstrated a decrease in TBUT and Schirmer-1 test scores and an increase in OSDI scores in rosacea patients. The ACV, ACD, and ACA values were significantly lower in the study population. The comparison of the corneal densitometry values revealed that the densitometry measurements in all concentric zones, especially in central and posterior zones were higher, and corneal aberrometric values in the posterior surface of the cornea were lower in the rosacea group compared to healthy controls.

The etiology of rosacea is still unknown, however, an overactive immune system, heredity, environmental factors, microorganisms such as Demodex folliculorum and Helicobacter pylori or a combination of these factors are among the several hypotheses mentioned in the literature. It is a chronic inflammatory dermatosis predominantly of the cheeks, nose, chin, and forehead and is characterized by recurrent episodes of flushing, transient or persistent central facial erythema, phymatous changes, papules, pustules, and telangiectasia.^19^ In a meta-analysis by Barakji et al. in which more than 9000 patients were assessed regarding the frequency of rosacea subtypes, the largest proportion (57%) was erythematotelangiectatic rosacea.^20^ The most common subtype was also erythematotelangiectatic rosacea (70.4%) in the present study population. Around one-third of the patients confront potential sight-threatening ocular involvement causing varying degrees of ocular morbidity.^6^ In 2019, ROSacea COnsensus (ROSCO) panel was established to address the spectrum of clinical presentation to improve patient management.^21^ Previous studies investigated the ocular manifestations of rosacea consisting of dry eye, blepharitis, meibomian gland dysfunction, and ocular irritation alongside corneal properties such as corneal hysteresis and corneal resistance factor.^10^, ^22^, ^23^

Two major subtypes of DED consist of aqueous-deficient and evaporative are associated with reduced lacrimal gland function and accompanied mostly by meibomian gland dysfunction respectively. These subtypes are considered as a part of a spectrum rather than being distinct entities and coexist as a continuum.^24^ Previous studies reported lower TBUT and Schirmer-1 test scores in rosacea patients.^1^, ^10^ Lower interleukin and VEGF levels were also demonstrated in rosacea patients which may lead to inflammation on the ocular surface and thus result in a decrease in tear film function tests.^25^ In the present study, the authors also observed lower TBUT and Schirmer-1 scores in accordance with the aforementioned studies.

In a study by Cetin et al., an evaluation of corneal and anterior chamber morphology in patients with noninfectious intraocular inflammation revealed no statistical changes in CT, ACD, and ACV between patients with active or inactive uveitis and the control group.^26^ It is known that inflammation leads to obstruction of aqueous outflow either via the accumulation of inflammatory cells in the intertrabecular space, edema of the trabecular lamellae, angle closure due to ciliary body swelling or scar formation, or membrane overgrowth in the anterior chamber angle.^27^ The authors also observed decreased ACV, ACD, and ACA in the study population. On the other hand, an assessment of corneal properties in Rheumatoid Arthritis (RA) patients revealed no statistically significant difference in terms of keratometry, CoV, and ACD.^16^ Similarly, the authors did not observe any significant difference in terms of keratometry values (K1, K2, and Kmax), CT, and CoV in the study population.

Corneal Scheimpflug densitometry has gained ground recently as a consequence of noncontact scans of the cornea which are easy to perform, quick, repeatable, and not subject to variations between observers. Corneal densitometry provides quantitative measurements of corneal clarity and transparency leading to more reliable means of monitoring corneal pathology and interventions. The main sources of corneal light scattering are the corneal epithelial layer and corneal endothelium. To the best of the authors’ knowledge, this is the first study that evaluated the corneal densitometric and aberrometric alterations in rosacea patients. Koh et al. reported increased corneal backward light scattering in patients with dry eyes.^15^ In addition, another study demonstrated higher corneal densitometry values in RA patients compared to healthy controls.^16^ Furthermore, a study by Cetin et al. which was conducted in a pediatric study population with inflammatory disease revealed higher corneal optical density in children who had uveitis.^28^ Similarly, corneal densitometry values in most of the concentric zones in all layers were higher compared to the control group in the presentstudy. Pentacam Scheimpflug imaging system also calculates the anterior and posterior corneal Zernike coefficients based on corneal elevation data. In a study by Yildirim et al., the RMS of total, LOAs, HOAs, and spherical aberrations were significantly higher in dry eye patients and authors concluded that artificial tears reduced the anterior corneal aberrations.^17^ The present results revealed similar findings regarding RMS of total, LOAs, and HOAs.

The present study has its limitations. Relatively small sample size, variable time interval to hospital admission, and lack of follow-up data are among the limitations of the study. Even though none of the patients had keratitis, conjunctivitis, or uveitis, the authors included all rosacea patients without classifying who had ocular rosacea and who did not. Future studies with larger sample sizes may also enlighten the mechanisms of controversial anterior segment findings by evaluating rosacea patients who have uveitis and those who do not.

## Conclusion

This study demonstrates the ocular manifestations of rosacea including DED, corneal properties, and anterior segment alterations. Given the fact that ocular signs may precede cutaneous disease, rosacea is frequently underrecognized by ophthalmologists. Therefore, a comprehensive examination of the ocular surface and assessment of the anterior segment is essential. The main priority of the ophthalmologist is to treat meibomian gland dysfunction and Demodex infection to prevent undesired ocular outcomes. The role of inflammation in the pathogenesis of rosacea may be better understood by prospective clinical studies with larger sample sizes.

## Financial support

None declared.

## Authors’ contributions

Erman Bozali: Approval of the final version of the manuscript, critical literature review, data collection, analysis and interpretation, effective participation in research orientation, intellectual participation in propaedeutic and/or therapeutic management of studied cases, preparation and writing of the manuscript, study conception and planning.

Duygu Yalınbaş Yeter: Approval of the final version of the manuscript, critical literature review, data collection, analysis and interpretation, effective participation in research orientation, intellectual participation in propaedeutic and/or therapeutic management of studied cases, manuscript critical review, preparation and writing of the manuscript, statistical analysis, study conception and planning.

Mustafa Tosun: Approval of the final version of the manuscript, data collection, analysis and interpretation, effective participation in research orientation, intellectual participation in propaedeutic and/or therapeutic management of studied cases, manuscript critical review, preparation and writing of the manuscript, statistical analysis.

Anıl Selim Apa: Approval of the final version of the manuscript, data collection, analysis and interpretation, effective participation in research orientation, intellectual participation in propaedeutic and/or therapeutic management of studied cases.

## Conflicts of interest

None declared.
